# Supplementary material for: Long-term high-grain diet altered the ruminal pH, fermentation, and composition and functions of the rumen bacterial community, leading to enhanced lactic acid production in Japanese Black beef cattle during fattening
Source: PLoS One. 2019 Nov 26;14(11):e0225448. doi: 10.1371/journal.pone.0225448 (PMC6879135; doi:10.1371/journal.pone.0225448)
Supplement: Attachment — Submitted filename: #5 Authors Responses to Reviewers_Rev1-1 (JB, PLOS).docx [file pone.0225448.s001.docx]

PONE-D-19-20947

Long-term high-grain diet altered the ruminal pH, fermentation, and composition and functions of the rumen bacterial community, leading to enhanced lactic acid production in Japanese Black beef cattle during fattening

Dear Editor and Reviewers

Authors would like to thank Editor and Reviewers for their helpful comments and suggestions. We have done our best to address all the issues raised by Reviewers very carefully in this first revision, which we believe has improved the quality of the paper further. To facilitate the reviewing process, we have highlighted all changes done by Authors (Yellow) in the revised manuscript. We have responded to every comment done by the Reviewers below, and also have indicated the changes made with respective new lines.

Before proceeding the present revision, we would like to declare that we have two corresponding authors who contributed equally to this work. The relevant changes are declared in the cover letter and title page of the manuscript.

5. Review Comments to the Author

Please use the space provided to explain your answers to the questions above. You may also include additional comments for the author, including concerns about dual publication, research ethics, or publication ethics. (Please upload your review as an attachment if it exceeds 20,000 characters)

Reviewer #1: General comments

The paper intitled ‘Long-term high-grain diet altered the ruminal pH, fermentation, and composition and functions of the rumen bacterial community, leading to enhanced lactic acid production in Japanese Black beef cattle during fattening’ described rumen characteristics, such as pH, VFA, and lactic acid, blood metabolites, and rumen microbial abundance and diversity of Japanese Black beef cattle during Early, Mid and Late fattening stages. The manuscript is well-prepared. The novelty about the work lies on the evaluation of rumen characteristics of cattle which are fed high levels of concentrate from 10 to 30 months, and how animals can cope the diet.

**AU:** Authors would like to thank Reviewer 1 for your helpful comments and suggestions. We have done our best to address all the issues raised by Reviewer 1 very carefully in this new revision, which we believe has improved the quality of the paper further.

The study has one major flaw which should be addressed. According to experimental design described, animals were on considered on Early fattening stage from 10 to 14 months. However, animals were fistulated when they were 12 months old. Thus, considering at least on month for recovery in the best case scenario, and another 3 weeks for diet adaption, different fattening stages should be considered.

**AU**: We would like to thank you for your comment. As you mentioned, animals were fistulated when they were 12 months old. After the surgery by skilled veterinarian, calves suffered from temporal loss of appetite, but no longer than 2 or 3 days, and no apparent adverse effects of cannulation were observed as similar to previous report (Kristensen et al., 2010; Technical note: Ruminal cannulation technique in young Holstein calves: Effects of cannulation on feed intake, body weight gain, and ruminal development at six weeks of age) although there is differences in age (6 weeks vs. 12 months of age) and breed (Holstein vs. Japanese Black cattle). Furthermore, no sign of obvious illness and antibiotic use was observed during the experimental period as mentioned in the original manuscript L363-365, which also are added in the Result section L220-221. Therefore, the effect of surgery was minimized in the present study, and no additional dietary adaptation was needed because Early fattening stage diet was fed before 2 months before surgery (10 months of age). Even if considering at least one month for recovery in the best case scenario and another 3 weeks for diet adaption, we considered that calves were fully adapted to Early stage diet during 13 to 14 months of age.

Also, minor considerations should be the forage-to-concentrate ratio that was modified throughout the experiment, being 26:74 during Early stage, 13:87 during the Middle stage, and 14:86 during Late stage, which might have affected the rumen environment and pH values. Also, the diet composition should be presented, and the methods used for chemical analysis. Vitamin A concentration should also be included in the Table 1, as you discussed hypovitaminosis in the Introduction section.

**AU**: We would like to appreciate for your comment. As you mentioned, the diet composition was modified throughout the experiment period, and it affected the rumen environment and pH values. During the fattening period, dietary modification according to different fattening stage is a common practice in Japanese Black cattle (original manuscript L343-345), and we followed general agreement in Japan (research and commercial farm) as introduced previously (Oka et al., 1998; Ogata et al., 2019). Furthermore, the present experiment presupposed dietary modification during the entire fattening stages to explore long-term changes in ruminal pH and fermentation, as well as their consequences with respect to the rumen bacterial community (original manuscript L345-347). However, we would like to apology for our mistake that the forage-to-concentrate ratio in the original manuscript was based on the intake amount, and it is revised accordingly in the revised manuscript L89-90 as “The forage-to-concentrate ratio was gradually decreased from 38:62 to 22:78, 13:87 to 10:90 and 8:92 to 7:93 during the Early, Middle, and Late stages, respectively”. In addition, chemical composition of the diets was analyzed according to the official method analysis of the Association of Official Analytical Chemists (AOAC) that registered in the Official Method Feed Analysis of Japan (MAFF, 2008) as we mentioned in the revised manuscript L98-101. Regarding the vitamin A concentration in the diet, we agree with your comment that it should be included in the Table 1. However, because vitamin A concentration is not directly related to our experiment, we did not measure dietary vitamin A concentration, and it can be predicted by peripheral blood concentration.

Finally, the lack of mechanical lysis of rumen content is troublesome and data interpretation and extrapolation should be made carefully. Several studies have shown that disruption of bacteria with tough cell walls is more efficient with a mechanical approach than by an enzyme-based protocol. Furthermore, extraction methods have important implications on the results, and studies using different extraction procedures should not be compared. For that, the conclusion needs to be re-worked.

**AU**: We would like to appreciate for your comment, and partly agree with your comment about mechanical lysis of rumen content. As you mentioned, DNA extraction methods and sampling techniques may affect the rumen microbial community structure (Henderson et al., 2013; Effect of DNA extraction methods and sampling techniques on the apparent structure of cow and sheep rumen microbial communities). In our study, total bacterial DNA was extracted as described previously Morita et al. (2007; An improved DNA isolation method for metagenomics analysis of the microbial flora of the human intestine) with minor modifications. Morita et al. (2007) reported that the improved DNA extraction method using lysozyme, proteinase K, and achromopeptidase gave stable and high-level lysis (>90%) for all the human fecal samples compared to the reference method (13.3-84.6%) and QIAamp DNA stool mini kit (38.8-69.2%). In addition, our study aimed to explore changes in the rumen fluid bacterial community after filtering through two layers of cheesecloth, and nearly no rumen contents are included in the fluid sample, possibly suggesting mechanical lysis of rumen contents has not much meaning in the present study. Unfortunately, to the best of our knowledge, we could not find studies that used enzymatic DNA extraction method in our field. Therefore, our DNA extraction method may reflect nearly true genomic information in the rumen microbial flora, while there are not many citable references in this kind of study.

Despite these considerations, the paper is well-prepared. Studies exploring the rumen microbiome and metabolic disorders are needed. If necessary, I would be available to look at the revised version.

**AU**: Authors would like to gratefully appreciate Reviewer 1 for your helpful comments and suggestions. Regarding studies exploring the rumen microbiome and metabolic disorder, a sentence about health problems induced by SARA is added in the revised manuscript L360-362 as “The occurrence of SARA may cause various health problems in cattle, such as feed intake depression, reduced fiber digestion, milk fat depression, diarrhea, laminitis, liver abscesses, increased production of bacterial endotoxins, and inflammation (Plaizier et al., 2008)”.

Specific comments

Please, verify financial disclosure guidelines and amend if appropriate. According to PLOS ONE guidelines, Funded studies should have statements with the following details: Initials of the authors who received each award; Grant numbers awarded to each author; The full name of each funder; URL of each funder website; and whether the sponsors or funders play any role in the study design, data collection and analysis, decision to publish, or preparation of the manuscript.

**AU**: We would like to appreciate for your comment. However, we do not have any grant information in this study. Therefore, we did not declare grant information in the manuscript.

P02L26: what ‘specialized high-concentrate diets’ means?

**AU**: In the present study, high-concentrate diets were not commercially available merchandise, but specially designed for the experimental cattle based on the Japanese Feeding Standard for Beef cattle. The relevant description is revised accordingly as “Specially designed high-concentrate diet” in the revised manuscript L26 and L86.

P02L29: the term ‘were collected on day 4 of pH measurement’ is confusing.

**AU**: In the present study, ruminal pH was measured during the final 7 days (days 1-7) of each fattening stage as described in the manuscript L101. The sentence “were collected on day 4 of pH measurement” is revised more in detail as “were collected on day 4 (fourth day during the final 7 days of pH measurement) during the ~” in the revised manuscript L29-30, L113-114, and L186-187.

P02L45-46: I respectfully disagree with the first sentence of the Introduction section, as pasture based diet can also promote growth, productivity, and high-quality meat or milk.

**AU**: We would like to appreciate for your comment on our thought. We completely agree with your comment, and people, including us, easily overlook the importance of forage and the relevant things that the ruminants live on pasture. Recently, however, high-concentrate based diet is well known for high-energy property and used broadly because of a human’s purpose in the world. Therefore, the sentence is revised accordingly as “A high-grain based diet is essential for beef and dairy cattle, to maximize growth, productivity, and high-quality meat or milk.” in the revised manuscript L46-47.

P04L48-50: there are parts of the manuscript which are confusing, such as the phrase ‘As a result, ruminal pH decreases; subacute ruminal acidosis (SARA) and ruminal acidosis (RA) are defined by ruminal pHs of ≤ 5.6 and below, respectively (Nagaraja and Titgemeyer, 2007).’. For example, I would suggest modifying to ‘ruminal pHs values of ≤ 5.6’.

**AU**: We would like to thank you for your comment. The relevant sentence is revised according to your comment in the revised manuscript L49-51.

P05L69-71: this phrase should be re-worked as you did not evaluate the ‘effect of ruminal pH, bacterial community and fermentation on the fattening of 10-month-old Japanese Black beef cattle’. Rather, this work characterized ruminal pH, bacterial community and fermentation characteristics on different ages of Japanese Black beef cattle.

**AU**: We would like to appreciate for your comment. The relevant sentence is revised accordingly in the revised manuscript L70-71 as “we explored the effects of the ruminal pH, bacterial community and fermentation characteristics on different ages of Japanese Black beef cattle”.

P05L71: ‘this findings’ is incorrect.

**AU**: We would like to apology for our careless mistake. This sentence is revised accordingly in the revised manuscript L72 as “these findings”.

P06L82: what percentage of refusals?

**AU**: We would like to thank you for your comment. During the Early stage, a fixed amount of concentrate and forage diets were offered, and feed refusal rate of concentrate and forage diets were 12.6% and 12.2%, respectively as revised in the revised manuscript L88-89. However, diets were fed ad libitum during the Middle and Late stages, and the percentage of refusal was not accessed during these stages.

P06L83: why 10–14, 15–22, and 23–30 months of age were selected as different stages? It seems arbitrary. This should be addressed.

**AU**: As we mentioned above, there is general agreement in Japanese Black cattle fattening stage, which included Early, Middle, and Late stages (10–14, 15–22, and 23–30 months of age, respectively). Although specific months of age is not documented, many research groups and commercial farms followed this guideline as reported previously (Oka et al., 1998; Ogata et al., 2019; Maeda et al., 2019; Effect of feeding wood kraft pulp on the growth performance, feeding digestibility, blood components, and rumen fermentation in Japanese Black fattening steers). Therefore, we followed general agreement in Japan as introduced previously (Oka et al., 1998; Ogata et al., 2019), and this information is added in the revised manuscript L84-86 as “The fattening stages included Early, Middle, and Late stages (10–14, 15–22, and 23–30 months of age, respectively) according to general agreement in Japan (Oka et al., 1998; Ogata et al., 2019)”.

P06L89: the rationale to feed concentrate after 1 hour of forage should be addressed. Also, how concentrate availability was ensured?

**AU**: Generally, cattle consumes concentrate diet first, and then, access to forage diet latter. Under our high-grain diet feeding condition, the cattle may consume excessive amount of concentrate diet and suffer from severe depression of ruminal pH, such as acute ruminal acidosis. Therefore, to prevent possible disorder, we decided to offer forage diet in advance to concentrate diet. The relevant information is added in the revised manuscript L94-96 as “concentrate diet was supplied 1 h after a forage diet feeding to maximize forage diet intake and to prevent excessive consumption of concentrate diet during the Early stage”. In addition, diets were offered individually to each cattle, and daily intake amounts of concentrate and forage were also recorded individually. Therefore, there was no problem in concentrate diet availability during the entire study period.

P06L93: the term ‘sufficient rate’ is dubious. Use other term.

**AU**: We would like to apology for our dubious term. At first, we intended to describe how much offered feed can cover an expected daily weight gain in the experimental cattle. However, we choose inappropriate term in the relevant description as “sufficient rate”, and thus, we revise this description accordingly as “Nutrient adequacy rate” in the revised Table 1. Furthermore, supplementary description is added in the footnote as “Nutrient adequacy rate was based on the nutrient requirement of Japanese Feeding Standard for Beef Cattle (National Agriculture and Food Research Organization (NARO), 2009), with an expected daily weight gain of 0.8, 0.65, and 0.7 kg during the Early, Middle, and Late stages, respectively”.

P06L93: please, refer the Japanese feeding standard.

**AU**: We would like to apology for our careless mistake. The reference is added accordingly in the revised manuscript L101-102 as “The adequacy rate of diet was calculated based on the nutrient requirement of Japanese Feeding Standard for Beef cattle (NARO, 2009)”

P08L104: the phrase ‘Rumen fluid samples were collected on day 4 of the pH measurements during the Early…’ is confusing and should be re-worked.

**AU**: As we mentioned above, this sentence “were collected on day 4 of pH measurement” is also revised more in detail as “were collected on day 4 (fourth day during the final 7 days of pH measurement) during the ~” in the revised manuscript L113-114.

P09L122: there are two references of Kim et al. 2016, which should be differentiated according to PLOS ONE manuscript preparation guidelines.

**AU**: We would like to apology for our careless mistake. The references are distinguished accordingly (Kim et al., 2016a; Front Microbiol, and Kim et al., 2016b; Physiol Genomics) in the revised manuscript.

P09L128: what was the ratio of phenol/chloroform/isoamyl alcohols?

**AU**: The ratio of phenol/chloroform/isoamyl alcohols was 25:24:1, and the relevant information is added in the revised manuscript L137-138 as “with phenol/chloroform/isoamyl alcohol (25:24:1) (Wako Pure Chemical Industries Ltd.)”.

P09L134: authors need to clarify whether pyrosequencing was the used approach. Furthermore, this should be addressed throughout the manuscript.

**AU**: We would like to appreciate for your comment. In the present study, pyrosequencing approach was performed followed by manufacturer’s instruction (16S Metagenomic Sequencing Library Preparation; Preparing 16S Robosimal RNA Gene Amplicons for the Illumina Miseq System). Therefore, the information is added in the revised manuscript L144-145 as “Sequencing libraries preparation was performed according to the Illumina 16S Metagenomic Sequencing Library preparation guide (2013)”.

P11L175: the phrase ‘Rumen fluid samples were collected on day 4 of the pH measurements during the Early…’ is confusing and should be re-worked.

**AU**: As we mentioned above, this sentence “were collected on day 4 of pH measurement” is also revised more in detail as “were collected on day 4 (fourth day during the final 7 days of pH measurement) during the ~” in the revised manuscript L186-187.

Table 2: please, use ‘ab’ to indicate similarities between stages. For example, the minimum pH values should be presented as ‘5.43a, 5.30ab, and 4.98b’ for Early, Middle, and Late stages, respectively. This should be considered for other tables.

**AU**: We would like to appreciate for your helpful comment, and to apology for our careless mistake. As you mentioned, superscript ‘ab’ is added throughout the tables.

Table 3: data on acetic, propionic and butyric acids should be discussed in the text.

**AU**: We would like to thank you for your suggestion. In the present study, the proportions of these acids were not different throughout the fattening period, while they showed general feature of high-grain diet feeding in Holstein cattle as reported elsewhere (Khafipour et al., 2009; Nagata et al., 2018). Therefore, the data on acetic, propionic, and butyric acids are discussed in the Discussion section L383-385 as “Furthermore, decrease and increase in the proportions of acetic and propionic acids, respectively, were consistent with general feature of high-grain diet feeding in Holstein cattle (Khafipour et al., 2009; Nagata et al., 2018)”.

P26L349: the effect of feed intake reduction in the Late stage should be discussed. Did animals decrease feed intake, and reduced concentrate intake by 20% to mitigate health problems?

**AU**: Throughout the study period, animals did not show any health problems, such as high-temperature, dehydration, and diarrhea. In addition, there was no abnormal or critical (within physiological range) changes in blood metabolites during the fattening stages, and thus, we could not suggest any plausible answer to the reason. In the revised manuscript, these information are clarified in the Result (L220-221) and Discussion (L363-365) sections, and decreased feed intake is discussed in the Discussion (L367-368).

P27L385: the discussion on reduction of ruminal bacterial richness but not bacterial diversity, should not be limited to one study. Please expand this discussion using other studies that corroborate with you study, other which does not, as it is central to your work. I would also consider bacterial and sequencing limitations, as bead beating was not used.

**AU**: We would like to appreciate for your helpful comment. We also agree with your comment that discussion should be expanded using other studies that corroborate with our study. In the original manuscript of the Discussion section L396-399, we mentioned that “bacterial diversity and richness indices, correlations of pH parameters with bacterial diversity and richness were generally positive; low ruminal pH leads to low bacterial diversity and richness (Khafipour et al., 2009; Mao et al., 2013; Plaizier et al., 2017; Nagata et al., 2018)”. However, to our best knowledge, this is the first study demonstrating the relationship between the long-term high-grain diet feeding and bacterial diversity or richness. Therefore, we clarify this in the revised manuscript L405-407 as “To best our knowledge, this is the first study demonstrating the relationship between the long-term high-grain diet feeding and bacterial diversity or richness, and suggests that ~”.

P28L401-405: the different core microbiota observed in your study compared to the literature was expected. A diet enriched in concentrate was used, having greater levels compared to dairy cattle diets. Furthermore, dairy cattle is anatomically different to beef cattle, which has implication of passage rate for example, and passage rate has huge influence on the rumen microbiota. Finally, generally beef cattle is fed to maximize productivity and to produce beef with quality. On the other hand, dairy cattle are fed to maximize productivity, but considering health and reproductivity. Thus, nutrient requirements and managements are different.

**AU**: We would like to appreciate for your comment. Regarding the relevant differences in core microbiota in our study to Holstein cattle, we also agree with your comment. Therefore, the manuscript is revised accordingly to include your comment as “~ the genus Prevotella was generally the most predominant in the rumen bacterial community of Holstein cattle (Mao et al., 2013; Golder et al., 2014; Kim et al., 2016a; Nagata et al., 2018). This is because different breeds of cattle may have different feed passage rate through the digestive tract due to different eating and rumination behaviors (Aikman et al., 2008), and Holstein cows are fed high-grain based diet to maximize productivity, but considering health and reproductivity (Roche, 2006), compared with those fed to maximize productivity and to produce beef with quality (Ogata et al., 2019).” in the revised manuscript L425-431.

Reviewer #2: Line Comment

**AU:** Authors would like to thank Reviewer 2 for your helpful comments and suggestions. We have done our best to address all the issues raised by Reviewer 2 very carefully in this new revision, which we believe has improved the quality of the paper further.

29 It this total VFA concentration?

**AU**: We would like to apology for our ambiguous description. The description is revised accordingly as “total VFA production” in the revised manuscript L41.

72 Perhaps “understand” could be changed to “the understanding of”.

**AU**: We would like to appreciate for your suggestion. We revise the expression according to your suggestion as “the understanding of” in the revised manuscript L73-74.

81, 374 It is noted that the cattle were rumen-fistulated at 12-mnths of age, during the Early stage of the trial. Please speculate whether this would have influenced the results obtained during that phase. It was also noted that fistualtion was said to be done at 10-months of age on line 374. If cattle were fistulated at 10 months, no discussion of affects on the animals is necessary.

**AU**: We would like to thank you for your comment. As you mentioned as a response to Reviewer 1, no apparent adverse effects of cannulation were observed in the present study. After the surgery at 12 months of age by skilled veterinarian, calves suffered from temporal loss of appetite, but no longer than 2 or 3 days, and no apparent adverse effects of cannulation were observed as similar to previous report (Kristensen et al., 2010; Technical note: Ruminal cannulation technique in young Holstein calves: Effects of cannulation on feed intake, body weight gain, and ruminal development at six weeks of age) although there is differences in age (6 weeks vs. 12 months of age) and breed (Holstein vs. Japanese Black cattle). Furthermore, no sign of obvious illness and antibiotic use was observed during the experimental period as mentioned in the original manuscript L363-365, which also are added in the Result section L220-221. Therefore, we believe that the effect of surgery was minimized in the present study.

85, 88, 90, etc. Suggest using either “roughage” or “forage” for that portion of the diet.

**AU**: We would like to appreciate for your suggestion. “roughage” is revised to “forage” throughout the revised manuscript.

93, Table 1 What is “sufficient rate”? Is this a requirement? If it is a requirement, why are the units in “%”? Are the dairy intakes in kg of DM? Please clarify. Also, please supply a citation for the Japanese feeding standard.

**AU**: We would like to apology for our dubious term. As we responded to Reviewer 1, we intended to describe how much offered feed can cover an expected daily weight gain in the experimental cattle. However, we choose inappropriate term in the relevant description as “sufficient rate”, and thus, we revise this description accordingly as “Nutrient adequacy rate” in the revised Table 1. Furthermore, supplementary description is added in the footnote as “Nutrient adequacy rate was based on the nutrient requirement of Japanese Feeding Standard for Beef Cattle (NARO, 2009), with an expected daily weight gain of 0.8, 0.65, and 0.7 kg during the Early, Middle, and Late stages, respectively”. Regarding the Japanese Feeding standard, reference is added accordingly in the revised manuscript L101-102 as “The adequacy rate of diet was calculated based on the nutrient requirement of Japanese Feeding Standard for Beef cattle (NARO, 2009).”

117 Was the supernatant or the pellet analyzed for LPS activity? I ask because the LPS is presumably associated with the microbes and likely would be largely with the 11,000 x g pellet.

**AU**: We would like to thank you for your comment. In the present study, rumen fluid samples were centrifuged and supernatant and pellet were separated. Then, LPS activity was assayed using supernatant to minimize interruption of gram-negative bacterial cell membrane. Therefore, we clarify that supernatant LPS activity was assayed using a kinetic Limulus amebocyte lysate assay as “~ and supernatant LPS activity was assayed using a kinetic Limulus amebocyte lysate assay ~” in the revised manuscript L126-127.

213-217 Again, please clarify what is meant by “rates”.

**AU**: We would like to apology for our undescriptive description. As we mentioned above, “sufficient rate” is revised to “nutrient adequacy rate” in the Table 1, and thus, the relevant description “rate” is revised in detail as “Nutrient adequacy rates” in the revised manuscript L225.

Table 2 Why are there no superscripts on several of the values for the Middle treatment (as is seen in Table 4)? Normally, one would expect that, if those values were not different from Early and/or Late, they would share the superscript with the Early and/or Late. Sometimes the Middle values are intermediate, or even greater than, either Early and Late.

**AU**: We would like to appreciate for your helpful comment, and to apology for our careless mistake. As you and Reviewer 1 mentioned, superscript ‘ab’ is added throughout the tables.

Table 3 Why are there no superscripts for A/P ration for the Late treatment?

**AU**: We would like to appreciate for your helpful comment, and to apology for our careless mistake. As we mentioned above, superscript ‘ab’ is added throughout the tables.

383-385 Do you mean to say that the risk of SARA during the Early stage was low, because pH was actually higher during this stage?

**AU**: In the present study, ruminal pH during the Early fattening stage was higher than other stages. Although the duration of time where pH < 5.6 was not long to diagnose as SARA during the Early stage, ruminal pH value was already low to induce low bacterial diversity from the beginning of the fattening stage when compared with previous study (Nagata et al., 2018). Also, the depression was getting more severe during the latter period, resulting in reduced rumen bacterial richness but not bacterial diversity. Therefore, our conclusion was that the risk of SARA during the Early stage was “high”, because pH was actually “low” during the Early stage although we did not diagnose SARA based on the duration of time in the present study.

Additional comment

Normally, one would expect to see a table of diet composition, showing the feedstuffs used to construct the 3 diets fed in this trial. The authors might consider adding a table containing this information to the paper.

**AU**: We would like to thank you for your suggestion. During the three different fattening period, the cattle were fed simply two kind of diets, concentrate (specially designed for the experimental cattle) and rice straw. Therefore, forage in the Table 1 is revised in detail as “Rice straw” in the revised manuscript, and we consider that this can explain the feedstuffs used to construct the 3 diets in this study.
