# Supplementary material for: Long-term high-grain diet altered the ruminal pH, fermentation, and composition and functions of the rumen bacterial community, leading to enhanced lactic acid production in Japanese Black beef cattle during fattening
Source: PLoS One. 2019 Nov 26;14(11):e0225448. doi: 10.1371/journal.pone.0225448 (PMC6879135; doi:10.1371/journal.pone.0225448)
Supplement: Attachment — Submitted filename: #5 Authors Responses to Reviewers_Rev2-1 (JB, PLOS).docx [file pone.0225448.s002.docx]

PONE-D-19-20947R1

Long-term high-grain diet altered the ruminal pH, fermentation, and composition and functions of the rumen bacterial community, leading to enhanced lactic acid production in Japanese Black beef cattle during fattening

Dear Editor and Reviewers

Authors would like to appreciate Editor and Reviewers for your helpful comments and suggestions. We have done our best to address all the issues raised by Reviewer #1 very carefully in this second revision, which we believe has improved the quality of the paper further. To facilitate the reviewing process, we have highlighted all changes done by Authors (Yellow) in the revised manuscript. We have responded to every comment done by the Reviewer #1 below, and also have indicated the changes made with respective new lines.

6. Review Comments to the Author

Please use the space provided to explain your answers to the questions above. You may also include additional comments for the author, including concerns about dual publication, research ethics, or publication ethics. (Please upload your review as an attachment if it exceeds 20,000 characters)

Reviewer #1: Comments to the Author

I appreciate the explanations and revisions of the authors and believe, I can provide better comments and suggestions.

**AU**: Authors would like to thank Reviewer #1 for your helpful comments and suggestions. We have done our best to address all the issues raised by you very carefully in this new revision, which we believe has improved the quality of the paper further.

L83: please provide the body weight of animals prior to the experiment;

**AU**: We would like to appreciate for your comment. The body weight of animals prior to the experiment (10 months of age) is added in the revised manuscript L92-94 as “The mean ± SE body weight of the cattle was 335 ± 4.4, 439 ± 7.6, 562 ± 11.6, and 712 ± 18.5 kg on prior to the experiment (10 months of age), and Early (14 months of age), Middle (21 months of age), and Late (29 months of age) fattening stage sampling days, respectively.”.

L86-91: the concentrate composition is still missing. There are several considerations about animal performance, and the reluctancy to include feed composition troubles me. I understand that authors might have another paper with performance and other data and if so, please discuss accordingly in this manuscript. I also understand the performance was not the objective of this manuscript. However, intake and performance are central to discuss rumen microbiome modulation. For example, according to the provided body weights, animals gained 122 kg from Early to Middle, with an average daily gain of 0.51, which is 21.79 % lower from the expected daily weight gain. Furthermore, animals had an average daily gain of 0.63 kg and the estimation was 0.7 kg, thus animals performed 10.12 % less than what was expected for the Late stage. Moreover, feed intake decreased by 18% (Table 1) from Middle to Late stages. Yet animals had a gain in average daily weight. What is the rationale of this phenomenon?

**AU**: We would like to apology for our missing data. The concentrate composition is added in the revised manuscript L87-90 as “The concentrate diet was composed of barely, steam-flaked corn, wheat bran, and soybean meal and contains 71.2% total digestible nutrient (TDN) and 15.7% crude protein (CP), 72.2% TDN and 13.9% CP, and 72.8% TDN and 12.0% CP during the Early, Middle, and Late stage, respectively.”

Regarding the animal performance, we partly agree with your opinion that intake and performance are central to discuss rumen microbiome modulation. In the present study, actual daily gain of cattle was observed only 2 weeks of period at each stage (from 1 week before to 1 week after sample collection day), and average daily gain was 0.75 (93.8% than calculated amount for daily gain), 0.69 (106.2%), and 0.46 (65.7%) kg/day during the Early, Middle, and Late stages, respectively. In addition, average daily gain during the entire experimental period was 0.63 kg/day in the present study. However, as you mentioned, the performance was not the main objective of the present study. Furthermore, the Japanese Feeding Standard for Beef cattle aimed to increase intramuscular fat accumulation (highly marbled meat) as discussed in the Introduction and Discussion sections, which may not fit the general farming object in other countries that maximize growth performance of beef cattle. We also agree with the fact that feed intake is undoubtedly important to meet energy requirement for production. However, other factors, such as peripheral hormones, environment, and genetic strain, also significantly affect the animal performance. As an example, peripheral blood vitamin A level is known for modulating intramuscular fat deposition, and the fattening cattle are generally fed high-grain, low vitamin A-containing diets to induce greater intramuscular fat deposition, leading to highly marbled meat during the fattening period (Oka et al., 1998) as introduced in the Introduction (L62-66) and Discussion (L355-357) sections. Therefore, we considered that descriptions in our manuscript is sufficient to concisely discuss and suggest our study aim. Although our description is somewhat unclear to elucidate the rationale of the phenomenon, we alternatively added our opinion on the dietary intake in the revised manuscript L365-367 as “Dietary intake amounts were highest during the Middle stage and lowest during the Late stage as an adaptation to long-term high-grain diet feeding or response to significantly lowered ruminal pH during the latter fattening stage”.

L85-88: please provide the concentrate composition rather the phrase ‘specially designed high-concentrate’;

**AU**: We would like to apology for our missing data. As we mentioned above, concentrate composition is added in the revised manuscript L87-90. Please see the revised manuscript.

L89: authors included new values for forage-to-concentrate ratio. My consideration about forage-to-concentrate ratio was that it was ‘modified throughout the experiment, being 26:74 during Early stage, 13:87 during the Middle stage, and 14:86 during Late stage, which might have affected the rumen environment and pH values’. Forage-to-concentrate ratio was properly presented. However, the increase percentage of concentrate in the diet and how it affected the rumen microbiome was not satisfactorily discussed in the previous version of the manuscript. I would suggest author to keep forage-to-concentrate ration as it was in the first version, or clarify if the new value is the recommend values according to practices in Japan;

**AU**: We would like to thank you for your valuable suggestion, and the relevant description about forage-to-concentrate ratio is reversed as it was in the original manuscript. Regarding the increase percentage of concentrate in the diet and how it affected the rumen microbiome, the dietary composition (e.g. forage-to-concentrate ratio) was changed monthly during the experiment period as revised in the previous revision despite it is reversed to the original description. However, rumen fluid sample was collected only once at the end of each fattening period, and sample collection is not accord with the dietary compositional changes (monthly dietary change vs. fattening periodic sample collection). As a result of feeding management and as we presented in the manuscript, total VFA concentration decreased, and lactic acid concentration increased during the Late stage as the adaptation or response to long-term high-grain diet feeding and long-term high-grain diet compositional change. Although we fully agree with your valuable suggestion on further discussion on the dietary change and rumen microbiome, we believe that our data connected long-term fattening diet feeding to ruminal pH, rumen fermentation, bacterial community composition and structure, blood metabolites, their relationships, and their consequences to leading enhanced lactic acid production are presented accordingly in the present study.

L89-91: I would consider being more precise in the description of gradually decreased. Was it a weekly adjustment for example?

**AU**: We would like to appreciate for your comment. However, as you mentioned above, the relevant description is reversed as it in the original manuscript. Please see the revised manuscript L91-92.

L91: the body weight should be 562 instead of 561. According to data on table 1, the weight was 561.8, thus it should be rounded up accordingly;

**AU**: We would like to apology for our careless mistake. The body weight is revised accordingly as 562 in the revised manuscript L92.

L96-97: do authors collected feed intake for the whole trial, or only during the sampling weeks? If that was the case, please address possible limitations of this approach in the discussion section. For example, animals could have suffered acute feed intake by the end of the animal trial, which help explain intake and performance data;

**AU**: We would like to appreciate for your helpful comment. In the present study, feed intake amount was collected daily throughout the experiment period. In addition, we checked that there was no acute increase or decrease in feed intake amount throughout the study period. Therefore, we added the information in the revised manuscript L364 as “no clinical sign of abnormal body condition, such as high body temperature, acute feed intake, dehydration ~”.

Table 1: please indicate if data for daily intake amount is based on organic or dry matter.

**AU**: We would like to apology for our undetailed description. In the Table 1, daily intake amount was based on the organic matter, and it is added in the footnote of the Table 1.

L143;160;161: as I understand, you used the Illumina platform, which is based on reversible dye-terminator instead of pyrosequencing. Usually the pyrosequencing was employed using the Roche 454 sequencing platform. Please, address this accordingly;

**AU**: We would like to appreciate for your comment. The description is revised accordingly in the revised manuscript L145 (Library preparation and DNA sequencing), 161 (Sequencing data analyses), and 162 (All sequencing reads were~).

L364-365: since body temperature, dehydration and diarrhea were considered clinical signs, please include how these were monitored in the Material and Methods sections;

**AU**: We would like to appreciate for your suggestion. The relevant information is added in the revised manuscript L97-98 as “Abnormalities of body condition (body temperature, appetite, hydration, and defecation) were observed daily throughout the study period”.

L366-368: in the Late stage animals experienced rumen pH value under 5.6 for almost 11.5 hours, decreased VFA production, increased lactic acid, LPS, and aspartate transaminase concentration, which suggests SARA and decreased feed intake. However, check your data on feed intake and forage-to-concentrate ratio;

**AU**: We would like to appreciate for your comment. In the original manuscript, we interpreted the relevant results with caution to avoid any overspeculation beyond our study result and aim. Therefore, as you mentioned above, we carefully suggest the reasons for decreased feed intake during the Late stage in the revised manuscript L365-367 as “Dietary intake amounts were highest during the Middle stage and lowest during the Late stage as an adaptation to long-term high-grain diet feeding or response to significantly lowered ruminal pH during the latter fattening stage”.

Table 5: Please, verify if differences between stages are correct for the OTU8. Should Late and Middle stage be similar?

**AU**: We would like to appreciate for your comment and apology for our careless mistake on the statistical analysis. As you mentioned, we checked all the validity of data, and found statistical mistakes only in the Table 5. Therefore, we revised the relevant descriptions in the Result (L311-324) and superscripts in the Table 5. However, these revisions have any influence on other part of the manuscript.
